# Molecular Characterization and Antibiogram of *Acinetobacter baumannii* Clinical Isolates Recovered from the Patients with Ventilator-Associated Pneumonia

**DOI:** 10.3390/healthcare10112210

**Published:** 2022-11-03

**Authors:** Mohd Saleem, Azharuddin Sajid Syed Khaja, Ashfaque Hossain, Fahaad Alenazi, Kamaleldin B. Said, Soha Abdallah Moursi, Homoud Abdulmohsin Almalaq, Hamza Mohamed, Ehab Rakha

**Affiliations:** 1 Department of Pathology, College of Medicine, University of Hail, Hail 55476, Saudi Arabia; 2 Department of Medical Microbiology and Immunology, RAK Medical and Health Sciences University, Ras Al Khaimah P.O. Box 11172, United Arab Emirates; 3 Department of Pharmacology, College of Medicine, University of Hail, Hail 55476, Saudi Arabia; 4 Hail Health Cluster, King Khalid Hospital, College of Pharmacy, King Saud University, Riyadh 55421, Saudi Arabia; 5 Department of Anatomy, College of Medicine, University of Hail, Hail 55476, Saudi Arabia; 6 Laboratory Department, King Khalid Hospital, Hail 55421, Saudi Arabia; 7 Clinical Pathology Department, Faculty of Medicine, Mansoura University, Mansoura 7650030, Egypt

**Keywords:** *Acinetobacter baumannii*, XDR, IMP-1, VIM-2, NDM-1, VAP, ICU, hospital-acquired infections

## Abstract

A 2-year prospective study carried out on ventilator-associated pneumonia (VAP) patients in the intensive care unit at a tertiary care hospital, Hail, Kingdom of Saudi Arabia (KSA), revealed a high prevalence of extremely drug-resistant (XDR) *Acinetobacter baumannii*. About a 9% increase in the incidence rate of *A. baumannii* occurred in the VAP patients between 2019 and 2020 (21.4% to 30.7%). In 2019, the isolates were positive for IMP-1 and VIM-2 (31.1% and 25.7%, respectively) as detected by PCR. In comparison, a higher proportion of isolates produced NDM-1 in 2020. Here, we observed a high proportion of resistant ICU isolates towards the most common antibiotics in use. Colistin sensitivity dropped to 91.4% in the year 2020 as compared to 2019 (100%). Thus, the finding of this study has a highly significant clinical implementation in the clinical management strategies for VAP patients. Furthermore, strict implementation of antibiotic stewardship policies, regular surveillance programs for antimicrobial resistance monitoring, and screening for genes encoding drug resistance phenotypes have become imperative.

## 1. Introduction

Ventilator-associated pneumonia (VAP) is defined as pneumonia that emerges after 2–3 days or thereafter following the endotracheal intubation procedure and is responsible for nearly 50% of all hospital-acquired pneumonia cases. It is characterized by a new or progressive infiltrate, signs of systemic or deep infection, changes in sputum quality, and detection of an etiological agent [1]. It has been reported as one of the top listed and most frequent intensive care unit (ICU)-acquired infections, with an incidence range of 6 to 52% [2]. On clinical assessment, the discrimination of the causative agents and etiological conditions associated with VAP is difficult, as their occurrence shows a great diversity of geographical prevalence [3,4]. Nevertheless, VAP is still reported to be the main cause of morbidity and mortality, and is a significant economic burden [5].

Since the emergence of SARS-CoV-2 in 2019, numerous studies have been reported worldwide with a high incidence of carbapenem-resistant *A. baumannii* among the COVID-19 patients admitted to ICUs [6]. In the ICU, *Acinetobacter baumannii* has been reported as one of the most common bacteria responsible for severe hospital-acquired infection, with a high mortality rate ranging from 45% to over 80% when the causative infectious agent is extensively drug-resistant (XDR) [7]. Similarly, much fewer therapeutic options are available with multi-drug resistant (MDR) *A. baumannii* isolates. Hence, controlling the spread of *A. baumannii* is a significant obstacle as it survives on inanimate objects, such as endotracheal tubes and catheters, making them an important source of hospital-acquired infections [8].

The complex interplay between the endotracheal tube, various risk factors, the virulence traits of the etiological agent (mainly bacteria), and the host immune response are primarily responsible for VAP development. In ICU patients developing VAP, the presence of an endotracheal tube is the most important risk factor, resulting in the destruction of natural defense mechanisms (the cough reflex of the glottis and larynx) against microaspiration around the cuff of the tube [1]. The infectious agents obtain direct access to the lower respiratory tract through different mechanisms, including microaspiration, production of a biofilm laden within the endotracheal tube by bacteria (mainly gram-negative bacteria), the accumulation and exuding of secretions around the cuff, and compromised state of mucociliary clearance [9,10].

*Acinetobacter baumannii’s* biofilm-producing ability, an important virulence factor, is believed to be the main cause of its ubiquitous distribution in these extreme conditions [11]. In addition, many studies have shown biofilms’ role in overcoming the host defense mechanism against *A. baumannii* [1]. This study aimed to evaluate the risk factors and the role of *A. baumannii* in the development of VAP in ICU patients using molecular characteristics and antimicrobial resistance profiling.

## 2. Materials and Methods

### 2.1. Study Setting

The study was conducted at the Department of Pathology, Division of Microbiology, University of Hail, KSA. The patients were recruited from the ICU of King Khalid Hospital, Hail, KSA, from January 2019 to December 2020. Patients with VAP were included in this study. Patients’ characteristics are presented in Table 1.

### 2.2. Ethics and Consent

After obtaining approval from the Ethics Committee, Research Deanship, University of Hail (H-2020-236, letter number 23561/5/42; IRB Registration Number with KACS: H-08-L-074), the study was performed, patients were informed about the research, and informed consent was taken from them.

### 2.3. Specimen Collection and Processing

A total of 124 ICU patients with pneumonia or pneumonia-like symptoms were considered for analysis and identification of the clinical isolates. All possible respiratory samples were collected, including sputum, broncho-alveolar lavage (BAL) fluid, endotracheal aspirate (ETA), a swab from endotracheal intubation, or pleural fluid [1,8,10]. All the specimens were collected with mandatory aseptic precautions and sent to the microbiology lab for analysis and identification without any delay. The clinical samples were cultured on 5% blood agar and MacConkey agar and incubated overnight (16–18 h) at 37 °C in an incubator. A direct Gram-stained smear was made from all samples and examined under a Bright field microscope for preliminary identification. The quality of the sputum sample was checked by examining the smear at a low power field, and >25 pus cells/low power field or <10 squamous epithelial cells were accepted for culture analysis [12].

The clinical isolates were identified by conventional microbiological methods [13]. Further, the isolates were confirmed by BD Phoenix M50 (BD Diagnostic Systems, Oxford, UK), which simultaneously performs identification (ID) and antibiotic susceptibility testing (AST). The combination panel (ID/AST combo panel; one for gram-negative and one for gram-positive bacteria) consists of 2 sides. The identification contained 51 microwells with dried substrates, whereas the AST side contained 85 microwells with different antibiotics at serial two-fold dilutions. Identification was based on conventional, chromogenic, and fluorogenic reactions. The AST was based on turbidimetry and redox reactions to determine each antibiotic’s minimal inhibitory concentration (MIC).

### 2.4. The Antimicrobial Susceptibility Testing

#### Kirby–Bauer Disk Diffusion Method

Antimicrobial susceptibility testing was done by the Kirby–Bauer disk diffusion method [8,14,15,16] according to the Clinical and Laboratory Standards Institute (CLSI) recommendations 2020 [17]. A total of 21 antibiotics were used, including amikacin (30 µg), cefepime (30 µg), colistin (10 µg), gentamycin (10 µg), ciprofloxacin (5 µg), meropenem (10 µg), ceftazidime (30 µg), ceftriaxone (30 µg), imipenem (10 µg), cefoxitin (30 µg), aztreonam (30 µg), tigecycline (15 µg), trimethoprim/sulfamethoxazole (1.25/23.75 μg), piperacillin-tazobactam (100 µg/10 µg), piperacillin (100 µg), teicoplanin (30 µg), amoxicillin/clavulanic acid (20 µg/10 µg), ertapenem (10 µg), cefuroxime (30 µg), ampicillin (10 µg), and levofloxacin (5 µg). The zone of inhibition diameter was noted and interpreted as sensitive or resistant, according to the CLSI guidelines 2020, except for colistin and tigecycline, for which CLSI guidelines are unavailable. Keeping the breakpoints of ≤2 as sensitive and ≥4 as resistant, the zone sizes of colistin in the disk diffusion test were taken as ≥11 as susceptible and ≤10 as resistant [18].

Tigecycline was interpreted as ≥16 mm sensitive and ≤12 mm resistant [19]. All the A. baumannii strains were resistant to at least three classes of antibiotics, including all penicillin and cephalosporins (including their inhibitor combinations), fluoroquinolones, and aminoglycosides and were defined as multi-drug resistant (MDR). Moreover, when these MDR strains are resistant to carbapenems, they are said to be extensively drug-resistant (XDR). Similarly, when XDR strains are resistant to polymyxins and tigecycline, they are denoted as pan-drug resistant (PDR) [18].

### 2.5. Phenotypic Detection of Carbapenemase Enzyme Production by Modified Hodge Test (MHT)

XDR and PDR strains were subjected to the Modified Hodge test (MHT). *Acinetobacter baumannii* strains showing positive results in the MHT test were considered to produce carbapenemase enzyme. The test was carried out as per CLSI 2020 guidelines [17]. A carpet culture of imipenem-sensitive *E. coli* ATCC 25,922 was made on the Mueller Hinton agar plate. Following this, an imipenem disk (10 μg) was inoculated in the center. A test strain was streaked as straight lines from the center to the periphery of the plate, along with positive and negative control (*Klebsiella pneumoniae* ATCC BAA-1705 as positive control and *K. pneumoniae* ATCC BAA-1706 as negative control). After 16–18 h of incubation, a clover leaf-like distorted zone of inhibition of the imipenem disk was produced by a test isolate and interpreted as a positive result [20,21,22,23].

### 2.6. Molecular Identification and Characterization of the Acinetobacter baumannii Clinical Isolates

#### 2.6.1. DNA Extraction

From pure isolates of *A. baumannii*, a subculture was made in trypticase soy broth (TSB) and incubated at 37 °C. DNA from fresh cultures was extracted using the QIAamp DNA Mini Kit (QIAGEN, Hilden, Germany). The DNA purity and quantity were assessed using a NanoDrop-1000 spectrophotometer (Thermo Fisher Scientific, Wilmington, NC, USA).

#### 2.6.2. Molecular Characterization of Antimicrobial Resistance Genes by PCR

According to a previously published protocol, Metallo-beta-lactamase (MBL) genes, blaVIM, blaIMP, and blaNDM were assessed [17]. All necessary primers for the study were obtained from Thermo Fisher Scientific company. For molecular characterization of the *A. baumannii* strains by multiplex PCR, firstly, pure growth of the test strain was obtained, and DNA was extracted by the alkaline lysis method. Three pairs of primers were obtained from Thermo fisher scientific company with a size range of 232–621 (Supplementary Table S1). The total amplification volume subjected to multiplex PCR was 50-µL with 2-µL of the sample (DNA). The mixture for detecting blaVIM, blaIMP, and blaNDM genes contained a 1× PCR buffer (Tris-HCL, KCl, MgCl2, Deoxynucleotide triphosphate, 10 μmol/L of each primer, and 2 U of AmpliTaq Gold Polymerase (Roche, Meylan, France). The following temperature protocol was followed to carry out amplification: 94 °C for 10 min and a total of 36 cycles of amplification consisting of 94 °C for the 30 s, 52 °C for 40 s, and 72 °C for 50 s, with 5 min at 72 °C for annealing. DNA fragments were analysed by electrophoresis in a 2% agarose gel at 100 V for 1 h in 1× TAE (40 mmol/L Tris–HCl [pH 8.3], 2 mmol/L acetate, 1 mmol/L EDTA) containing 0.05 mg/L ethidium bromide.

### 2.7. Statistical Analysis

All the statistical analyses were performed using Microsoft Excel and the statistical package SPPS (version 23, IBM, Armonk, NY, USA). The prevalence of unknown parameter(s) from the target population was estimated using a random sample. The results are presented in frequencies and percentages.

## 3. Results

### 3.1. Bacterial Isolates

**Figure 1:**
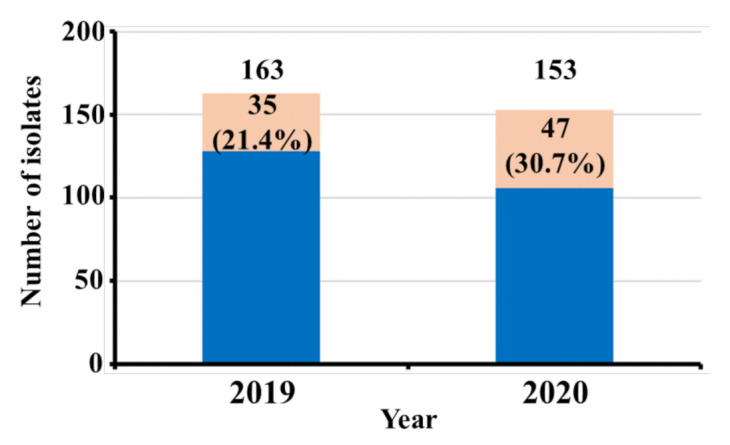
*A. baumannii* isolation rate among other isolates during the year 2019–2020.

The study was conducted from January 2019 to December 2020. A total of 591 patients were admitted to the ICU of King Khalid hospital in the year 2019, out of which only 163 patients’ samples were culture-positive with monomicrobial infection. Out of 163 clinical isolates, 35 isolates were *A. baumanniii*, giving a prevalence rate of 21.4%. Similarly, in the year 2020, a total of 487 patients got admitted, out of which only 153 patients’ samples were culture-positive with monomicrobial infection. Out of 153 clinical isolates, 47 isolates were identified as *A. baumannii*, giving a prevalence rate of 30.7% (Figure 1). The infection rate was higher in males than in female patients, with a ratio of 2.7 (60 males and 22 females. Most of the isolates were of XDR phenotypes as per the XDR definition proposed by ECDC (European Centre for Disease Prevention and Control) and CDC (Centre for Disease Control and Prevention). PCR-based gene detection was carried out to determine the prevalence of blaNDM, blaVIM, and blaIMP genes.

### 3.2. Antibiotic Susceptibility Testing

As shown in Table 2, *A. baumannii* isolates were resistant to the most common antibiotics used to treat common bacterial infections. In 2019, absolute resistance was seen for piperacillin-tazobactam, meropenem, imipenem, ciprofloxacin, aztreonam, cefepime, amoxicillin/clavulanic acid, cefoxitin, ertapenem, cefuroxime, ampicillin, levofloxacin, and ceftriaxone. In 2020, the same resistance pattern was observed with few changes; one isolate (2.1%) was sensitive for each piperacillin-tazobactam, meropenem, imipenem, and levofloxacin, whereas 5 isolates (10%) were sensitive for cefepime. The resistance pattern of bacterial isolates showed higher resistance towards most of the drugs in the year 2019. This scenario changed in 2020 as bacterial isolates exhibited less resistance to the antibiotics tested. The colistin sensitivity was higher (100%) in the year 2019 compared to the year 2020 (91.4%). A higher proportion of isolates were sensitive to aminoglycosides in 2020 compared to 2019. Absolute carbapenem resistance was seen in isolates during 2019 as compared to 2020 (4.2%). Teicoplanin was more sensitive in 2020 (19.1%) compared to 2019 (2.8% sensitivity). The same was observed for tigecycline, as its sensitivity rate was 14.8% in 2020 compared to 2.8% in 2019.

Out of the 35 *A. baumannii* positive isolates in 2019, more than half (51.4%) were from VAP patients (Table 3). Apart from VAP, the second most common infection was surgical site infection (SSI, 25.7%), followed by central-line associated bloodstream infection (CLABSI; 11.4%), catheter-associated urinary tract infection (CAUTI; 5.7%), bloodstream infection (BSI; 2.8%), and non-surgical site infection (non-SSI; 2.8%). In 2020, again, more than half of the *A. baumannii* positive isolates (25 out of 47; 53.4%) were from the VAP patients. *A. baumannii* positive isolates from non-SSI was 17% (8 out of 47), followed by SSI (12.7%, 6 out of 47). There was a 4.2% positive rate of *A. baumannii*, each from respiratory tract infection (RTI), CAUTI, CLABSI, and BSI (Table 3).

In our present study, we found a higher proportion of bacteria produced IMP-1 and VIM-2 (31.1% and 25.7%, respectively) compared to NDM-1 (8.5%) in 2019 (Table 4). In 2020, 14.8% of the bacteria produced NDM-1, which was higher than the percentage of the bacteria in 2019 (Table 4).

## 4. Discussion

VAP is a frequent hospital-acquired infection in severely ill patients encountered in mechanical ventilation cases. In the ICU, the second most common hospital-acquired infection in mechanically ventilated patients is shown to be VAP [24]. The VAP is associated with a prolonged hospital stay, mortality, healthcare expenses, and infection with MDR pathogens [25]. Early and late onset are the two types of VAPs. Although early-onset VAP (<5 d since hospitalization) shows a better prognosis with high susceptible bacteria, the late-onset VAP (>5 days since hospitalization) shows a poor prognosis with increased morbidity, mortality, and MDR pathogens. In our study, a higher percentage of antimicrobial resistance in ICU patients has been reported, similar to the findings of a previous Spanish study [26].

In the past two decades, *Acinetobacter* species have become increasingly common in ICUs, causing serious infections [27]. In our study, we found a constant association of *A. baumannii* with VAP for the years 2019 and 2020, which is 18 (51.4%) and 25 (53%), respectively. This high association of *A. baumannii* is also reported by several other studies [8,26,28]. We found a higher *A. baumannii* infection rate in males compared to female patients. This result is in agreement with the findings of other studies [29,30]. The colistin sensitivity was higher (100%) in the year 2019 compared to the year 2020 (91.4%). Based on several studies, including this study, colistin is the most effective antibiotic against drug-resistant isolates of *A. baumannii* [31,32,33]. In our study in the year 2019, we observed that all *A. baumannii* isolates were resistant to carbapenems, but on the other hand, they have shown 100% sensitivity to colistin. Hence, the absolute resistance toward carbapenems does not confer any adverse effect on the treatment therapy [26]. Similarly, in 2020, the same resistance pattern was seen with a drop in colistin sensitivity, i.e., 91.4% with 2.1% sensitivity towards carbapenems (imipenem and meropenem), which may be due to the diversity in the prevalence of the bacterial isolate. Aminoglycosides were more sensitive in the year 2020 as compared to the year 2019. With Teicoplanin, only 1 (2.8%) sensitivity was reported in 2019, whereas in 2020, the sensitivity percentage rose to 9 (19.1%).

In ICU, colistin-based combined therapies, colistin plus amikacin, are also used. As a result of the deadliest effect of combinatorial therapy, antibiotics exert a higher selective pressure on the gut flora than monotherapy, causing it to proliferate [34]. Furthermore, antibacterial combinations may expose users to additional risks [35]. We found that most of the clinical isolates were of XDR phenotypes. The higher prevalence of MDR and/or XDR strains of *A. baumannii* can be related to the irrational use of extended-spectrum antibiotics [36,37]. Resistance to broad-spectrum antibiotics is because of carbapenemase production by bacteria. The most common carbapenemase enzyme-producing genes are on mobile genetic elements and can frequently be passed on to other bacteria species [36,37]. Our study found that mostly VIM-1, IMP-2, and NDM-1 were prevalent, which is responsible for carbapenemase production and resistance against carbapenems. However, a study conducted by Asadian et al. reported that OXA-23 was the common gene responsible for carbapenemase production [38]. High mortality and limited treatment options are available for *A. baumannii*-associated VAP patients, as MDR is quite common in *A. baumannii* [39]. Another study by Nowak et al. from Greece in 2017 reported that OXA-23 is produced by 80% *A. baumannii* isolates from VAP [40]. In our current study, we noted that the most common infection associated with *A. baumannii* isolates was from VAP patients, and the prevalence rate was 51.3% and 53.4% for the years 2019 and 2020, respectively, which is similar to A. Chaari et al. (58.5%) [29]. As shown in Table 2, the isolated rate of *A. baumannii* from other infections was very low compared with VAP patients. In our study, we also reported a higher prevalence (25.7%) of *A. baumannii* isolates from SSI during the year 2019 as compared to the year 2020 (12.7%), as previously reported by Helal et al. (16.67%) in 2015 from SSI [41].

For patients with VAP caused by *A. baumannii*, the 2016 guidelines of the American Thoracic Society-Infectious Disease Society of America (ATS-IDSA) recommended the adoption of Polymyxins (Colistin or Polymyxin B) or Tigecycline [42]. Following increased Colistin application due to the emergence of MDR bacterial infections and VAP overseas [43], it is not recommended that colistin be used as first-line therapy for *A. baumannii*-associated VAP. The use of carbapenemase for strains susceptible to this drug should be continued. Imipenem shows superior bactericidal activity compared to colistin when treating pneumonia due to *A. baumannii* being considered [44].

## 5. Limitations, Benefits, and Future Approaches

The main limitation of this study is the limited number of strains included in it and the fact that detailed epidemiological characteristics of the strains were not recorded. The other limitation of the study is that there was a slight variation in how the sputum cultures were obtained from the patients, which may have affected the incidence rate of pneumonia among the patients. Studies have shown that obtaining sputum samples from upper or lower respiratory tract secretions might affect the positivity rate of pneumonia diagnosis [45,46,47]. The benefit of this study is that this is the first such study carried out with strains isolated at the King Khalid hospital of Hail, Saudi Arabia, and it is expected to increase awareness among physicians and researchers about the current status of this important pathogen. It will also help implement strict and effective antibiotic stewardship procedures. Future studies will focus on the detailed epidemiological characterization of the *A. baumannii* strains aiming to determine the possible clonality of the isolates.

## 6. Conclusions

The result of this study shows a higher association of *A. baumannii* with VAP, with high antibiotic resistance. Colistin still shows high sensitivity against XDR *A. baumannii* phenotypes, followed by aminoglycosides, Teicoplanin, and tigecycline. We found blaIMP and blaVIM as the most common genes responsible for resistance. Hence, strict antibiotic stewardship policies and regular surveillance programs for antimicrobial resistance should be used to reduce the emergence of drug-resistant *Acinetobacter baumannii* isolates. Genes responsible for drug resistance should be regularly monitored to determine the drug resistance trend.

## Figures and Tables

**Table 1:** Patients characteristics.

Characteristics	Mean (Min–Max)
**Age**	57.2 years (19–95)
**Sex**	
Male	61.20%
Female	38.80%
**BMI**	27.3 (17.8–38.2)
**Underlying disease/Diagnosis**	
Acute cardiovascular accident	17%
Septic Shock	12%
Hypertension, old CVA	11%
Acute respiratory failure	10%
Head Trauma	9%
Aspiration pneumonia	8%
Pneumonia	4%
Others	27%

**Table 2:** Comparison of antibiotics sensitivity pattern of *A. baumannii* isolated from 2019–2020.

S. No.	Antibiotics	2019—Total *A. baumannii* = 35 *n* (%)	2020—Total *A. baumannii* = 47 *n* (%)
1	Colistin (10 µg)	35 (100)	43 (91.4)
2	Amikacin (30 µg)	4 (11.4)	22 (48.9)
3	Piperacillin-Tazobactam (100 µg/10 µg)	R	1 (2.1)
4	Piperacillin (100 µg)	1 (2.8)	R
5	Gentamicin (10 µg)	4 (11.4)	8 (17)
6	Meropenem (10 µg)	R	1 (2.1)
7	Imipenem (10 µg)	R	1 (2.1)
8	Ciprofloxacin (5 µg)	R	R
9	Teicoplanin (30 µg)	1 (2.8)	9 (19.1)
10	Aztreonam (30 µg)	R	R
11	Cefepime (30 µg)	R	5 (10.6)
12	Ceftazidime (30 µg)	1 (2.8)	R
13	Amoxicillin/clavulanic acid (20/10 µg)	R	R
14	Trimethoprim/Sulfamethoxazole (1.25/23.75 µg)	2 (5.7)	R
15	Cefoxitin (30 µg)	R	R
16	Ertapenem (10 µg)	R	R
17	Cefuroxime (30 µg)	R	R
18	Ampicillin (10 µg)	R	R
19	Tigecycline (15 µg)	1 (2.8)	7 (14.8)
20	Levofloxacin (5 µg)	R	1 (2.1)
21	Ceftriaxone (30 µg)	R	R

**Table 3:** Comparison of *A. baumannii* isolated from different clinical infections from 2019 to 2020.

S. No.	Types of Infection	*n* (%)—2019	*n* (%)—2020
1	Ventilator-associated pneumonia	18 (51.4)	25 (53.2)
2	Catheter-associated Urinary Tract Infection	2 (5.7)	2 (4.3)
3	Central Line-associated Bloodstream Infection	4 (11.4)	2 (4.3)
4	Bloodstream Infection	1 (2.8)	2 (4.3)
5	Surgical Site Infection (SSI)	9 (25.7)	6 (12.8)
6	Non-SSI	1 (2.8)	8 (17)
7	Respiratory tract infections	--	2 (4.3)
	Total	35	47

**Table 4:** Distribution of genes among clinical isolates of *A. baumannii* during 2019–2020.

S. No.	Gene	*n* (%)—2019	*n* (%)—2020
1	bla_VIM-2_	9 (25.7%)	12 (25.5%)
2	bla_IMP-1_	11 (31.4%)	8 (17%)
3	bla_NDM-1_	3 (8.5%)	7 (14.8%)

## Data Availability

The original and raw data used and reported in this study are available from the first author and corresponding author.

## Supplementary Materials

The following supporting information can be downloaded at: https://www.mdpi.com/article/10.3390/healthcare10112210/s1, Table S1: Primers used in this study.
