# Supplementary material for: Molecular Characterization and Antibiogram of Acinetobacter baumannii Clinical Isolates Recovered from the Patients with Ventilator-Associated Pneumonia
Source: Healthcare (Basel). 2022 Nov 3;10(11):2210. doi: 10.3390/healthcare10112210 (PMC9690950; doi:10.3390/healthcare10112210)
Supplement: Supplementary file 1 [file healthcare-10-02210-s001.zip › healthcare-1843485-supplementary.pdf]

Table S1. Primers used in this study.

Primer	Sequence (5′-3')	Gene	Amplicon size (bp)
VIM-F	GATGGTGTTTGGTCGCATA	bla_{VIM}	390
VIM-R	CGAATGCGCAGCACCAG		
IMP-F	GGAATAGAGTGGCTTAAYTCTC	bla_{IMP}	232
IMP-R	GGTTTAAYAAAACAACCACC		
NDM-F	GGTTTGGCGATCTGGTTTTC	bla_{NDM}	621
NDM-R	CGGAATGGCTCATCACGATC		
